# Serum NFL and tau, but not serum UCHL-1 and GFAP or CSF SNAP-25, NPTX2, or sTREM2, correlate with delirium in a 3-year retrospective analysis

**DOI:** 10.3389/fneur.2024.1356575

**Published:** 2024-03-19

**Authors:** Johannes Heinrich Alexander Piel, Leon Bargemann, Frank Leypoldt, Klaus-Peter Wandinger, Justina Dargvainiene

**Affiliations:** ^1^Department of Neurology, University Hospital Schleswig-Holstein, Kiel, Germany; ^2^Institute of Clinical Chemistry, University Medical Center Schleswig-Holstein, Kiel, Germany

**Keywords:** delirium, Alzheimer’s disease, neurofilament light chain, tau protein, glial fibrillary acidic protein, neuronal pentraxin 2, single-molecule array, soluble triggering receptor expressed on myeloid cells 2

## Abstract

Delirium represents a common terminal pathway of heterogeneous neurological conditions characterized by disturbances in consciousness and attention. Contemporary theories highlight the acute impairment of synaptic function and network connectivity, driven by neuroinflammation, oxidative stress, and neurotransmitter imbalances. However, established biomarkers are still missing. Innovative diagnostic techniques, such as single-molecule array analysis, enable the detection of biomarkers in blood at picomolar concentrations. This approach paves the way for deeper insights into delirium and potentially therapeutic targets for tailored medical treatments. In a retrospective 3-year study, we investigated seven biomarkers indicative of neuroaxonal damage [neurofilament light chain (NFL), ubiquitin carboxyl-terminal hydrolase (UCHL-1), and tau protein], microglial activation [glial fibrillary acidic protein (GFAP) and soluble triggering receptor expressed on myeloid cells 2 (sTREM2)], and synaptic dysfunction [synaptosomal-associated protein 25 (SNAP-25) and neuronal pentraxin 2 (NPTX2)]. The analysis of 71 patients with delirium, Alzheimer’s disease (AD), and non-AD controls revealed that serum NFL levels are higher in delirium cases compared to both AD and non-AD. This suggests that elevated NFL levels in delirium are not exclusively the result of dementia-related damage. Serum tau levels were also elevated in delirium cases compared to controls. Conversely, cerebrospinal fluid (CSF) SNAP-25 showed higher levels in AD patients compared to controls only. These findings add to the increasing body of evidence suggesting that serum NFL could be a valuable biomarker of neuroaxonal damage in delirium research. Although SNAP-25 and NPTX2 did not exhibit significant differences in delirium, the exploration of synaptic biomarkers remains promising for enhancing our understanding of this condition.

## 1 Introduction

Delirium is defined as a disease characterized by rapid onset and fluctuating impairment in consciousness, attention, perception, memory, psychomotor activity, emotion, and circadian rhythm. It often represents a common end-stage manifestation of various neurologic syndromes (1), with phenotypic and etiological diversity. Delirium has long been attempted to be categorized into clinical phenotypes such as hypoactive, hyperactive, mixed, inflammatory, and non-inflammatory delirium. Hypoactive and mixed delirium in particular are less frequently recognized and are associated with a poorer prognosis (2). Despite its temporary nature, delirium can lead to cognitive impairment for up to 12 months (3). It poses an independent risk for increased morbidity and mortality rates, with a 1.5-fold risk of death within a year, following an episode and a 2- to 4-fold increase in the overall mortality rate in intensive care unit (ICU) settings (4). Hospitalized elderly individuals are particularly vulnerable, with the prevalence ranging from 29% to 64% and reaching up to 80% in postoperative settings, hospices, geriatric wards, and ICUs (5). Despite its widespread prevalence and significant impact, the pathophysiology of delirium is not well understood. Current theories propose a multifactorial, acute disruption of synaptic activity as the primary mechanism. The most common factors include neuroinflammation, neuronal aging, oxidative stress, disturbances in neurotransmitters, melatonin, and neuroendocrine systems, as well as network disconnectivity (6). Most models suggest an interplay of these factors. Its progression and prognosis are heavily influenced by the underlying conditions. The causes of delirium can be categorized into predisposing and precipitating factors. Predisposing factors, which lower the threshold for precipitating factors, include age, cognitive impairment, dementia, visual and hearing impairment, stroke, depression, and alcohol misuse (7). Precipitating factors encompass sepsis, trauma, surgery, anesthesia, polypharmacy, and metabolic changes (4). It has been proposed to categorize precipitating factors for delirium as hypoxic, septic, sedative/narcotic, metabolic, or unknown, with an unknown cause being associated with a poorer prognosis (8). However, multiple triggers may occur simultaneously, and lesser triggers are sufficient, particularly when there is susceptibility to predisposing factors. Collectively, these factors disrupt the balance of neurotransmitters and cytokines, trigger inflammation, and increase the permeability of the blood–brain barrier, leading to cognitive dysfunction and the onset of delirium (7). However, delirium is not merely a transient network dysfunction. It can lead to permanent neuronal loss and can mark the beginning of neurodegeneration (9), partly explaining the long-term consequences and its role as an independent risk factor for morbidity and mortality (4). Current treatments focus on addressing its precipitating factors, such as sepsis or metabolic changes, as well as sedative and antipsychotic symptomatic therapies. However, causative interventions for synaptic dysfunction are lacking, leaving the treatment of delirium predominantly symptomatic.

Recently, novel humoral markers of neurodegeneration, neuroinflammation, and synaptic dysfunction have gained attention as potential diagnostic and prognostic biomarkers in neurodegenerative diseases. Key among these are neurofilament light chain (NFL) (10, 11), ubiquitin carboxyl-terminal hydrolase (UCH-L1) (12), and total tau protein (tTau) (13–15), which account for markers of neuroaxonal damage and degeneration, followed by markers of neuroinflammation and astrocytic and microglia activation, such as glial fibrillary acidic protein (GFAP) (12, 16, 17) and soluble triggering receptor expressed on myeloid cells 2 (sTREM2) (18–20), as well as markers of synaptic function such as synaptosomal-associated protein 25 (SNAP-25) (21) and neuronal pentraxin 2 (NPTX2) (22–24). Most of these markers have been extensively studied and have demonstrated a correlation with disease severity in Alzheimer’s disease (AD) (25–27); however, their correlation has been studied to a lesser extent in delirium so far (28).

Recent findings indicate a correlation of serum NFL levels with delirium severity (10), with a robust correlation of cerebrospinal fluid (CSF) levels and plasma concentrations (29). Both total and phosphorylated tau levels have been linked to post-surgical delirium severity (30). GFAP has shown increased serum levels in conditions such as septic acute encephalopathy (12) and postoperative delirium (16). However, the correlation between GFAP and delirium remains ambiguous, with multiple studies reporting inconsistencies (10, 14). UCH-L1, part of the ubiquitin-proteasome system, is a marker for neuronal repair, and its decrease has been associated with protein-misfolding diseases such as AD and Parkinson’s disease (PD) (31). A soluble form, sTREM2, is considered to counterbalance TREM2 activity, thus being linked with proinflammatory microglial processes (18). One study has shown elevated CSF sTREM2 levels in patients with hip fracture surgeries who developed delirium, particularly in those without preexisting dementia (20). Increased levels of the synaptic protein SNAP-25, a component of the presynaptic soluble NSF attachment protein receptor (SNARE) complex, were associated with AD, PD, and Creutzfeldt–Jakob disease (CJD) (32). NPTX2, also known as neuronal activity-regulated protein, has been reported to have reduced CSF levels in various neurodegenerative diseases such as AD (22), genetic frontotemporal dementia (FTD) (23), and dementia with Lewy bodies (DLB) (24), correlating with cerebral atrophy and cognitive dysfunction (33). Both SNAP-25 and NPTX2 have not been studied in delirium yet.

In summary, markers of neurodegeneration, neuroinflammation, and synaptic function are not disease-specific, so changes may be related to both delirium and preexisting neurodegeneration, as neurodegenerative conditions are common in delirium. However, different biomarker-level compositions could expand our pathophysiological knowledge and pave the way for prospective studies. Therefore, in this present retrospective study, our objective was to determine whether patients with delirium exhibit significantly altered markers of neurodegeneration, neuroinflammation, and synaptic dysfunction compared not only to age-matched non-AD controls with neurological conditions that are not known to alter the biomarkers, such as normal pressure hydrocephalus, but also patients with AD. Furthermore, we hypothesized that disruption of synaptic function might be more important in delirium than neurodegeneration, which would be a major difference from dementia, leading to the potential utility of synaptic biomarkers as well as the ratio of neurodegeneration to synaptic dysfunction in differentiating delirium from dementia and other neurological conditions.

## 2 Materials and methods

The study and all its analyses adhered to the Declaration of Helsinki. All patients provided consent for the retrospective evaluation of their residual samples, and the ethics committee of University Hospital Schleswig-Holstein granted approval.

### 2.1 Patients and samples

We retrospectively recruited patients with delirium and two control groups: (1) patients diagnosed with AD and (2) age-matched non-AD controls with neurological conditions that were an indication for CSF puncture but have not been reported to lead to relevant changes in the investigated biomarkers. Patients were identified through ICD-10 codes (Supplementary Table 1) from 1 January 2017 to 31 March 2021, and samples were available from 7 November 2017 to 30 March 2021. The inclusion criteria for the study are as follows: (1) documented informed consent; (2) age over 60 years in the control group to align with the age range of AD and delirium groups; (3) availability of stored, matched CSF/serum samples; and (4) in the AD control group, typical CSF biomarker constellation, defined by a decreased CSF Aβ1-42/Aβ1-40 ratio and increased CSF phosphorylated tau (pTau_181_). However, biomarkers such as NFL or tTau are not disease-specific and have been found to be elevated in neurodegenerative diseases and after acute events such as stroke or cardiac surgery (34, 35). Although these events may precipitate delirium, they also increase biomarkers more than delirium itself. We excluded patients with (1) any acute neurological event (such as stroke, seizure, or encephalitis) or cardio/neurosurgical interventions within the past 12 months, (2) any neurodegenerative processes other than AD, (3) metastatic tumors or primary brain tumors, excluding grade 1 meningioma, (4) severe polyneuropathy (PNP), and (5) inflammatory CSF syndromes (>20 cells/μL). A thorough manual review of all available medical records was conducted to verify the conclusiveness of diagnoses and their temporal precedence over the acquisition of the stored blood samples. Patients in the delirium cohort were classified according to their discharge diagnosis of delirium, based on ICD-10 diagnostic criteria. The diagnosis of delirium was performed by neurology specialists and was coded in the discharge letter. However, protocols leading to the diagnosis were not further defined in the file, so the verifiability was critically assessed at the time of inclusion in the study on the basis of a thorough review of the medical file. The samples were collected between 0 and 12 days after the onset of delirium (Supplementary Figure 1). Patients in the AD control group were classified following the National Institute on Aging–Alzheimer’s Association (NIA-AA) research framework of 2018 (36). This framework categorizes AD based on three biomarker dimensions: amyloid pathology (A), tau hyperphosphorylation (T), and non-specific neurodegeneration (N). A diagnosis of AD requires biomarkers for both amyloid and tau pathologies (A+/T+). Non-specific neuronal injury (N) is assessed using either brain imaging techniques or tTau levels. Furthermore, patients were categorized by their clinical characteristics into preclinical AD, mild cognitive impairment (MCI), and dementia, using the McKhann criteria for AD dementia (37) and the Albert criteria for MCI due to AD (38).

Serum and CSF samples were frozen at −80°C in polypropylene tubes within a maximum of 48 h at 4°C after sample collection and were stored in the biobank under continuous monitoring of the freezer temperature.

### 2.2 Measurements of serum NFL, tau, GFAP, and UCHL-1

In this study, 71 serum samples were analyzed in duplicates using a commercially available 4-Plex A (NFL, tau, GFAP, UCHL-1, Quanterix, Billerica, Massachusetts, USA) assay with a single-molecule array (Simoa^®^) HD-X analyzer (Quanterix, Billerica, Massachusetts, USA) according to the manufacturer’s instructions, with intra- and inter-assay variance of <3.8% for NFL, 12.6% for tTau, 2.8% for GFAP, and 18.6% for UCHL-1. When the estimated concentration fell below the lowest calibrator value, a concentration of the lowest calibrator value was substituted. This occurred one time (1/142 single measurements) for NFL and GFAP and two times (2/142) for tTau, but 14 times (14/142 measurements) for UCH-L1, indicating a significant floor effect in the latter.

### 2.3 Measurements of CSF SNAP-25, NPTX2, and sTREM2

SNAP-25, NPTX2, and sTREM2 could be measured in 65 CSF samples. CSF levels of SNAP-25 were measured in duplicates using a commercially available SNAP-25 Advantage Kit assay with a single-molecule array (Simoa^®^) HD-X analyzer (Quanterix, Billerica, Massachusetts, USA) according to the manufacturer’s instructions with intra- and inter-assay variance of <3.4%.

CSF levels of NPTX2 and sTREM2 were measured in duplicates using commercially available NPTX2 and sTREM2 ELISA kits (Innotest^®^, Fujirebio, Tokyo, Japan) according to the manufacturer’s instructions, with intra- and inter-assay variance of <7.24 and 17.21%, respectively.

### 2.4 Statistical analysis

Statistical analysis was performed using Prism 9.2.0 (GraphPad) and IBM SPSS Statistics 29.0. For categorical data, Fisher’s exact tests were employed for group comparisons. Both age and biomarker levels demonstrated non-normal distributions, as indicated by a visual appraisal of QQ plots (Shapiro–Wilk test: p_age_ = 0.0022, p_NFL_ < 0.0001, p_tau_ < 0.0001, p_GFAP_ < 0.0001, p_UCHL-1_ < 0.0001, p_sTREM2_ < 0.0001, p_SNAP-25_ = 0.0006, p_NPTX2_ < 0.0001). Consequently, the Kruskal–Wallis one-way analysis of variance (ANOVA) was utilized for analyzing non-parametric data. Multiple regression analysis was carried out to determine if age and neurological comorbidities (specifically cortical atrophy, cerebral microangiopathy, and pre-established dementia) could predict the association between serum NFL levels and delirium. To achieve a normal distribution of residuals, serum NFL levels were log-transformed for multiple regression, and the regression coefficient β is expressed in logarithmic units of serum NFL. The efficacy of biomarkers predicting delirium was evaluated through receiver operating characteristic (ROC) analysis. A *p*-value of <0.05 was considered statistically significant.

## 3 Results

### 3.1 Demographics

**Figure 1:**
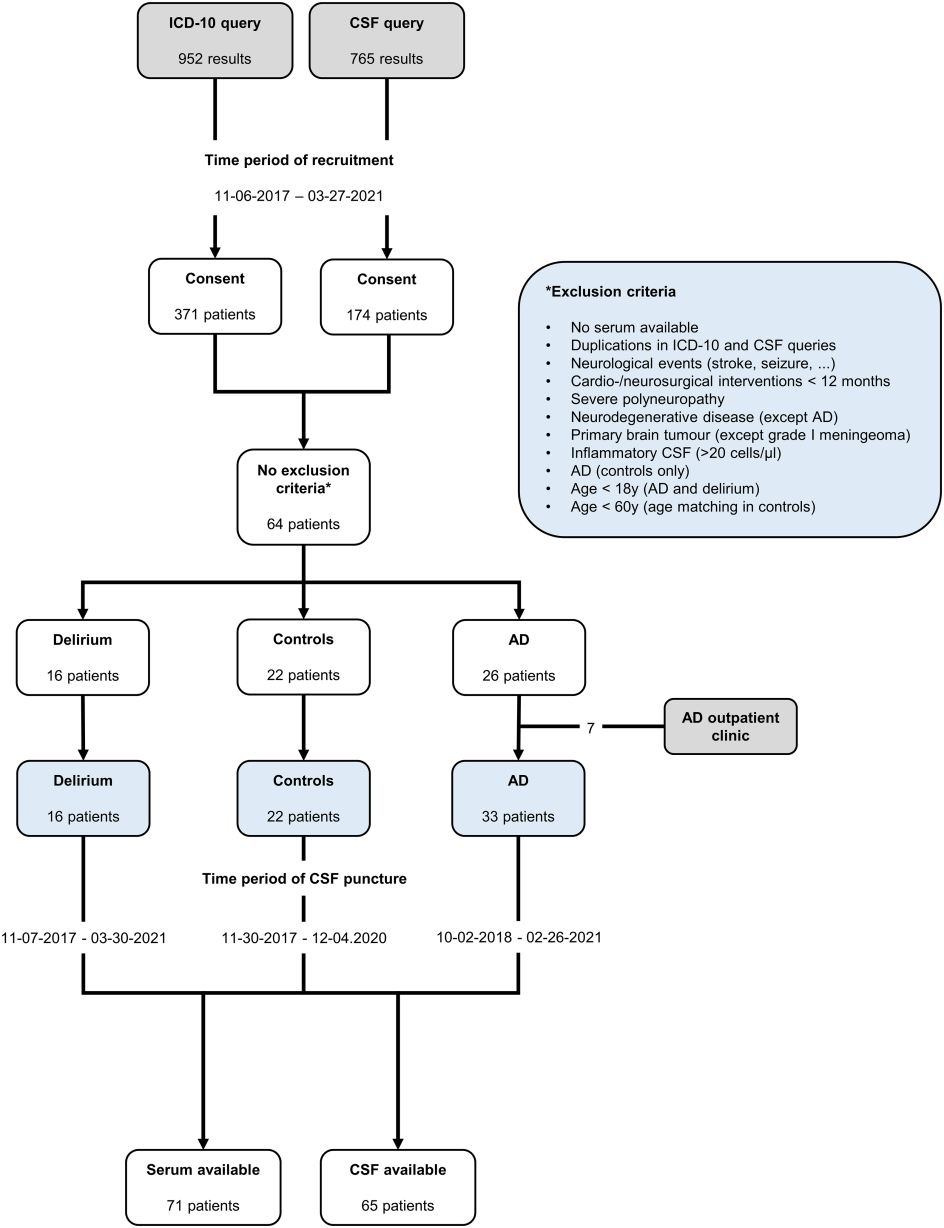
Inclusion diagram of retrospective patient recruitment.

A total of 16 patients with delirium, 33 with AD, and 22 non-AD controls were included in the analysis (Figure 1). Demographic details and comorbidities are presented in Table 1. The precipitating factors for delirium varied, including infection (four cases), hyponatremia (two), unknown causes (two), drug withdrawal (two), postoperative complications, opiate intake, hypertensive encephalopathy, syncope, prerenal renal failure, and changes in environment. Of the delirium patients, three experienced the onset during hospitalization, while the remaining 13 developed delirium in non-hospital settings, with most being admitted to the hospital on the same day or within a week after delirium onset (Supplementary Figure 1). Furthermore, 13 delirium patients were admitted to general neurological wards, of whom 3 were later transferred to intermediate care (IMC) units because of severe agitation caused by delirium, while 3 were directly admitted to IMC units because of severe agitation (one case), hypertensive urgency (one case), and infection-related delirium (one case). No patient was admitted to the ICU. After a median hospital stay of 9.5 days (IQR 6–13.5), 13 patients, who had recovered from delirium, were discharged. One patient passed away during palliative care, and two were transferred to psychiatric hospital for ongoing delirium treatment. In the delirium cohort, AD biomarkers were reported in the patient records of 11 out of 16 patients. Among these, one patient met the A/T/N criteria for AD but had not been diagnosed with dementia prior to admission. All but one patient with delirium received electroencephalograms (EEGs), which were normal or showed only generalized slowing and, in no case, regional slowing or epilepsy-type potentials. All delirious patients underwent cranial computed tomography (cCT), and seven received cranial magnetic resonance imaging (cMRI). Cerebral microangiopathy was present in 14 cases, cortical atrophy was present in 12 patients, and chronic stroke (no anamnesis, diagnosis, or imaging indicated events within the last 12 months) was found in 5 patients with delirium (Table 1; Supplementary Figure 1). Body mass index (BMI) was assessed in 5 out of 16 patients (median 27.46, 95% CI 22.31–31.11).

**Table 1:** Demographics and comorbidities.

	AD	Delirium	Controls	Significance
*N*	33	16	22	
Age	76 (73–79)	79.5 (74–87)	73 (64–79)	0.0759 (Kruskal–Wallis)
Median (95% CI)				
Sex	14/19	9/7	14/8	0.281 (Fisher)
M/F				
**Premorbid comorbidities**				
Neurologic	31 (94%)	16 (100%)	19 (86%)	0.256 (Fisher)
Cortical atrophy	28 (85%)	12 (75%)	4 (18%)	<0.001* (Fisher)
Microangiopathy	22 (67%)	14 (88%)	8 (36%)	0.004* (Fisher)
Pre-described dementia	4 (12%)	8 (50%)	0 (0%)	<0.001* (Fisher)
PNP	3 (10%)	4 (25%)	12 (54%)	<0.001* (Fisher)
Chronic stroke	5 (15%)	5 (31%)	1 (5%)	0.080 (Fisher)
Systemic infections	3 (9%)	7 (44%)	3 (14%)	0.017* (Fisher)
Cardiac	24 (73%)	14 (88%)	13 (59%)	0.153 (Fisher)
Pulmonary	3 (9%)	1 (6%)	3 (14%)	0.773 (Fisher)
Metabolic	17 (52%)	9 (56%)	7 (32%)	0.266 (Fisher)
Oncologic	12 (36%)	3 (19%)	3 (14%)	0.144 (Fisher)
Musculoskeletal	9 (27%)	7 (44%)	10 (45%)	0.331 (Fisher)
Others	13 (39%)	8 (50%)	9 (41%)	0.780 (Fisher)

*p < 0.05.

All patients in the AD cohort, as per inclusion criteria, exhibited a decreased CSF Aβ1-42/Aβ1-40 ratio and increased CSF pTau_181_ levels. Neuroimaging (cMRI where available, otherwise cCT) classified 29 patients as A+/T+/N+, and 4 patients were classified as A+/T+/N−. Among the 33 AD patients, 24 had dementia, 8 had MCI (prodromal AD), and 1 was classified as preclinical AD because there were only isolated deficits in divided attention in an otherwise unremarkable neuropsychological test with 138 points on the Mattis Dementia Rating Scale and normal findings in its subscales.

The primary diagnoses for the non-AD control group included normal pressure hydrocephalus (six cases), non-severe polyneuropathy (four), somatization disorder (three), small-fiber neuropathy (three), tension headache (two), multifactorial gait disorder, pseudodementia due to depression, disk bulging without myelopathy, and poly-arthrosis.

All patients in the study had comorbidities (Table 1). Infections such as sinusitis, pneumonia, urinary tract infection, chronic hepatitis C, and sepsis were significantly more prevalent in the delirium group compared to the AD or control cohorts (*p* = 0.017). Almost all patients had predisposing neurological conditions, including cortical atrophy, cerebral microangiopathy, previously diagnosed dementia, polyneuropathy (PNP), and stroke, even though patients with acute neurological events within the 12 months prior to inclusion were excluded. Newly diagnosed delirium and newly identified dementia were not considered preexisting neurological comorbidities, but known cases of premorbid dementia were included. Significantly higher occurrences of cortical atrophy (*p* < 0.001), cerebral microangiopathy (*p* = 0.004), and known premorbid dementia (*p* < 0.001) were found in the delirium and AD cohorts compared to the control group. Conversely, non-severe PNP was notably more common in the control group (*p* < 0.001). Beyond these findings, no significant differences were observed between the groups. Cardiac conditions, including arterial hypertension, atrial fibrillation, heart failure, coronary heart disease, and myocardial infarction, were frequently observed across all cohorts. Non-metastatic hematologic or oncologic conditions (such as monoclonal gammopathy of undetermined significance (MGUS), breast cancer, or prostate cancer) were not excluded from the study and showed no significant differences among the groups, similar to metabolic diseases such as diabetes, hypercholesterolemia, and electrolyte disturbances. Other occasional comorbidities, including psychiatric disorders (such as depression), gastrointestinal disorders, and chronic renal failure, were grouped into a single category.

### 3.2 Humoral biomarkers and delirium

**Figure 2:**
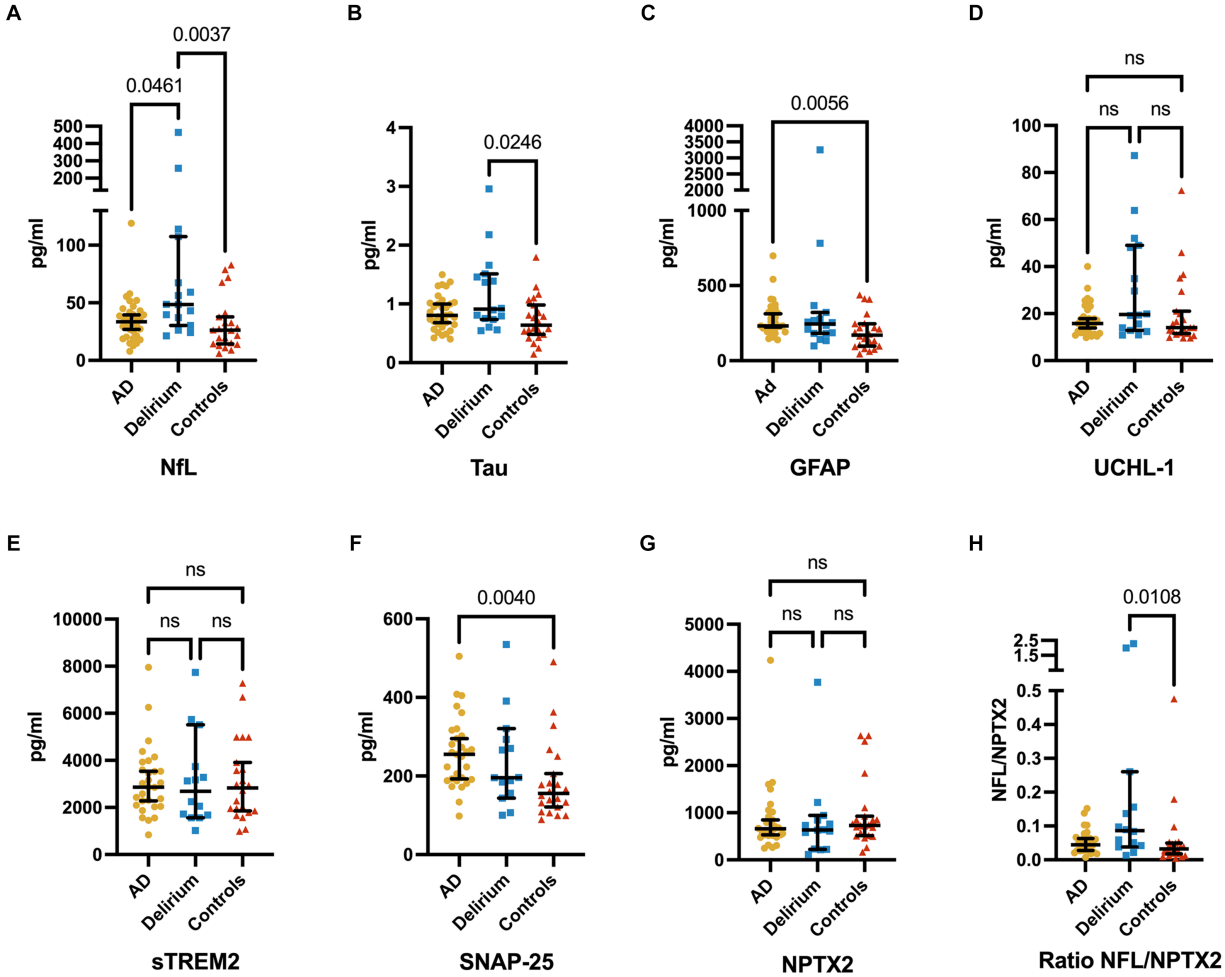
Serum levels of NFL **(A)**, total tau **(B)**, GFAP **(C)**, and UCHL-1 **(D)**, CSF levels of sTREM2 **(E)**, SNAP-25 **(F)**, and NPTX2 **(G)**, and the ratio of serum NFL and CSF NPTX2 **(H)** (median ± 95% CI). Serum levels of NFL are significantly higher in delirium patients compared to non-AD controls and AD, while serum tau levels are significantly higher in delirium patients compared to non-AD controls only. GFAP is significantly increased in AD patients compared to non-AD controls only. Furthermore, a trend of higher tau levels in the delirium cohort over the AD cohort is seen. No significant differences are found in UCHL-1 serum levels. CSF levels of SNAP-25 are significantly higher in AD patients compared to non-AD controls, and no significant differences are found in sTREM2 and NPTX2 CSF levels. Ratios of NFL and NPTX2 show significant differences only between delirium patients and non-AD controls. The *p*-values are corrected for testing three groups.

A total of 71 serum samples and 65 CSF samples were analyzed in this study. We found significantly higher serum NFL levels in patients with delirium compared to both the control group and, to a lesser extent, the AD cohort (Figure 2). Total serum tau levels were significantly elevated in delirium patients compared to non-AD controls, but not when compared to the AD group. Median serum GFAP and CSF SNAP-25 levels were higher in the AD cohort than in non-AD controls, but not in the delirium cohort. There were no significant differences among the three groups in serum UCH-L1 levels nor in CSF levels of sTREM2 and NPTX2.

Serum NFL remained significantly associated with the presence of delirium (compared to non-AD controls and AD combined) in a multiple regression, adjusting for the influence of age, cortical atrophy, cerebral microangiopathy, and previously diagnosed dementia (β_delirium_ = 0.8087, p_delirium_ = 0.0004, β_age_ = 0.0080, p_age_ = 0.4230, β_atrophy_ = 0.4943, p_atrophy_ = 0.0088, β_microangiopathy_ = −0.2661, p_microangiopathy_ = 0.1858, β_dementia_ = −0.2334, p_dementia_ = 0.3542, *R*^2^ = 0.2645; Supplementary Table 2).

There was no correlation between serum NFL or tTau levels and the sub-classification of AD (preclinical AD, prodromal AD [MCI], and apparent AD). Furthermore, across all three cohorts, only mild correlations were observed between serum (NFL, tTau, GFAP, UCHL-1) and CSF (SNAP-25, sTREM2, NPTX2) biomarkers (Spearman’s ρ ranging from 0.21 to 0.36). In contrast, strong correlations were noted between the three CSF biomarkers (Spearman’s ρ between 0.59 and 0.65; Supplementary Figure 2).

### 3.3 Biomarker combinations

Subsequently, we analyzed whether a ratio of neurodegeneration to synaptic dysfunction (specifically, NFL compared to SNAP-25 or NPTX2 and tau compared to SNAP-25 or NPTX2) would more effectively differentiate patients with delirium from control groups (AD and non-AD). Significant differences were observed in the ratios of NFL and NPTX2 in patients with delirium compared to non-AD controls, but not when compared to the AD group (Figure 2). However, no significant differences were noted in the ratios of NFL and SNAP-25, tau and SNAP-25, as well as tau and NPTX2.

**Figure 3:**
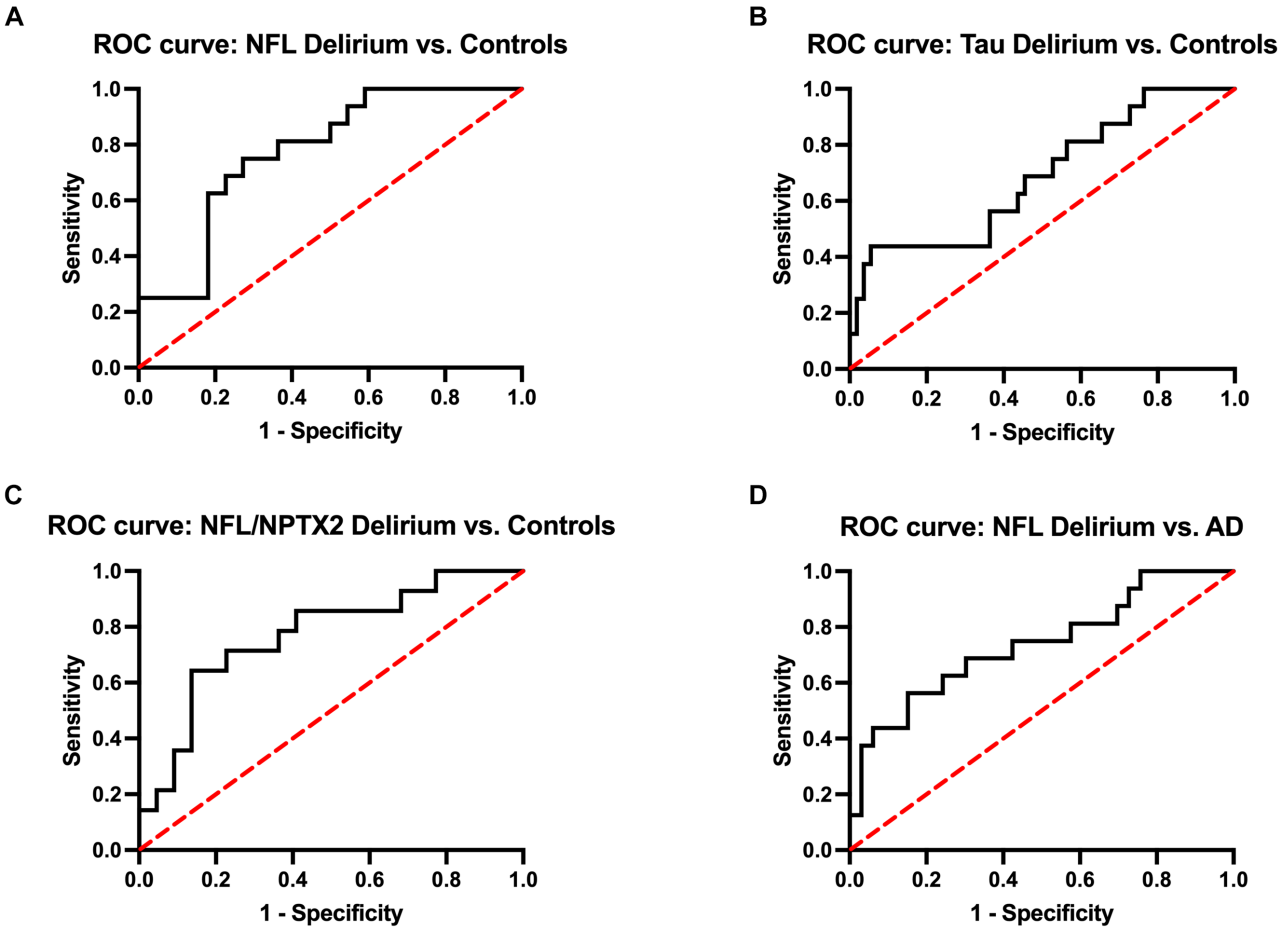
ROC curves of serum NFL **(A)**, serum tau **(B)**, and the ratio of NFL and CSF NPTX2 **(C)** to discriminate delirium from non-AD controls and ROC curve of serum NFL to discriminate delirium from AD **(D)**. The AUC values for distinguishing delirium from non-AD controls are 0.78 (95% CI 0.63–0.92) for NFL, 0.74 (95% CI 0.58–0.90) for tau, and 0.77 (95% CI 0.61–0.93) for NFL/NPTX2. The AUC for distinguishing delirium from AD is 0.74 (95% CI 0.58–0.89).

Additionally, the receiver operating characteristic (ROC) analysis was performed to assess the predictive accuracy of specific biomarkers for delirium (Figure 3). This analysis included serum levels of NFL and tTau, along with the ratio of serum NFL and CSF NPTX2, which were all significantly elevated in the delirium cohort compared to non-AD controls (Figure 2). The area under the curve (AUC) values for distinguishing delirium from non-AD controls were 0.78 (95% CI 0.63–0.92) for NFL, 0.74 (95% CI 0.58–0.90) for tTau, and 0.77 (95% CI 0.61–0.93) for NFL/NPTX2 ratio. According to the Youden index, the optimal cutoff points were 34.99 pg/mL for NFL (sensitivity 75%, specificity 73%), 0.60 pg/mL for serum tTau (sensitivity 88%, specificity 50%), and 0.06 for the NFL/NPTX2 ratio (sensitivity 64%, specificity 86%). Given the significant difference in serum NFL levels between the delirium and AD cohorts, an additional ROC analysis was performed to evaluate the effectiveness of NFL in differentiating delirium from AD. The optimal cutoff point was found to be 39.62 pg/mL (sensitivity 69%, specificity 70%) with an AUC value of 0.74 (95% CI 0.58–0.89).

## 4 Discussion

This retrospective study highlights three key findings: First, NFL levels showed the strongest correlation with delirium, AD, and control groups among seven biomarkers. Second, tTau levels were significantly higher in the delirium cohort compared to non-AD controls and were not significantly elevated in the delirium group compared to the AD group. Third, contrary to presumed role of synaptic dysfunction in delirium, SNAP-25 was only elevated in AD, but not in delirium. NPTX2 levels did not significantly differ across groups. Consequently, the ratio of neurodegeneration (represented by NFL) to synaptic dysfunction (represented by NPTX2) did not improve the differentiation of delirium from non-AD controls compared to NFL alone.

Research increasingly supports the role of neurodegenerative biomarkers in delirium. A recent prospective study on postoperative delirium identified elevated NFL levels but not tTau, GFAP, or UCHL-1 levels (10). Another study linked both tTau and, to a lesser extent, NFL levels to postoperative delirium severity and recovery, unlike GFAP (14). In addition, higher levels of NFL have recently been shown to be associated with cognitive decline in a 2-year follow-up study after acute illness and delirium (28). Plasma tTau, a non-specific marker for cognitive decline and gray matter atrophy in AD, may predict the development of MCI, especially in longitudinal analyses (39–41). In AD, the combination of tau hyperphosphorylation and Aβ aggregation is crucial to the pathophysiology and axonal decline. Phosphorylated tau is specific to tauopathies such as AD and might only hint toward an underlying AD pathology as a predisposing factor when examined in the context of delirium (42). Therefore, we did not investigate it in our study. Total tau, on the other hand, indicates general axonal loss. The increase in tTau levels in delirium might be due to neuroinflammation, as suggested by its correlation with interleukin-(IL)-8 and IL-10 (14).

NFL and tTau have been linked to critical illness in ICUs independent of an underlying neurological cause and showed correlation with both disease severity and clinical outcomes, for example, in the context of severe SARS-CoV-2 infections (43, 44). None of the delirium patients in our study were critically ill and were admitted to an ICU; however, it could be considered a potentially limiting factor for the clinical utility of these biomarkers since delirium is particularly relevant in ICU settings (4).

CSF levels of sTREM2, a marker linked to microglial neuroinflammation (18), were higher in patients developing delirium post-hip surgery (20), particularly in those without dementia. This suggests that the pathogenesis of delirium may differ in the presence of dementia (20). Our study did not observe elevated sTREM2 levels in delirium patients, half of whom had previously diagnosed dementia. Additionally, sTREM2 levels were reported to increase transiently, particularly before the onset of delirium (20). As the lumbar puncture in our study was performed post-diagnosis of delirium, this might explain our negative results. Thus, sTREM2 could signal delirium risk rather than serve as a diagnostic biomarker, as supported by another study showing higher serum sTREM2 levels in patients who later developed postoperative delirium (45).

One recent study noted increased GFAP levels in delirium patients after undergoing cardiac surgery (16). However, GFAP’s reliability as a biomarker is less established compared to NFL and tTau in delirium. Our research adds evidence of elevated NFL and tTau levels in delirium, suggesting that they are more pertinent than GFAP and UCHL-1. Additionally, our results show that these biomarker elevations are not just due to age or AD, highlighting the importance of axonal loss in the pathophysiology of delirium.

Synaptic dysfunction is considered to be crucial in the pathogenesis of delirium, as suggested by the ‘Cognitive Disintegration Theory’, which links delirium severity to neuroinflammation and neuronal connectivity (46–48). However, in our study, synaptic biomarkers were not correlated with delirium. SNAP-25 levels were elevated only in AD, known for neurodegeneration, congruent with previous reports of elevated levels in AD, PD, and CJD, among other fluid biomarkers (32). NPTX2 has similarly been shown to be elevated in AD, DLB, and FTD (22–24). While the absence of a correlation with these biomarkers in our study does not dismiss the role of synaptic dysfunction in delirium, it indicates the need to explore other biomarkers such as presynaptic proteins involved in vesicle release and postsynaptic receptors, such as neurogranin, synaptotagmin, synaptobrevin, syntaxin, growth-associated protein 43, α-synuclein, synaptic vesicle glycoprotein 2A, and other neuronal pentraxins (21, 32). This is of particular interest to further test the hypothesis that synaptic dysfunction may play a greater role in delirium compared to neurodegeneration and that synaptic biomarkers may be helpful in differentiating delirium from dementia.

Our study assessed the comorbidities across the three cohorts, finding significant differences in systemic infections and neurologic comorbidities (Table 1). Infections, which are known to trigger delirium (4), were significantly more common in the delirium cohort. A recent study on serum NFL levels in pneumonia linked NFL to encephalopathy but not to disease severity (49), indicating infections did not bias NFL levels in our delirium cohort. We observed more cortical atrophy and cerebral microangiopathy in both AD and delirium groups compared to non-AD controls, and pre-diagnosed dementia was more common in the delirium cohort compared to non-AD controls. Cortical atrophy, indicating neurodegeneration, is used in stratifying the neurodegeneration (N) in the A/T/(N) criteria for AD classification (36). However, it is not disease-specific (36). Interestingly, cortical atrophy has been linked to delirium in ICU patients, with cortical thinning observed in regions not typically associated with AD, suggesting distinct underlying pathologies (50). Cerebral microangiopathy, relevant in vascular dementia but also implicated in the pathophysiology of AD (51), has also been linked to delirium (52, 53). The complex interrelationship between delirium and dementia is evident; dementia predisposes delirium (7), and delirium may affect dementia progression, though the specifics have not yet been fully understood (54). Previous reports have suggested that these comorbidities might affect the NFL to some degree (55–57). To evaluate their impact on NFL levels, we included cortical atrophy, cerebral microangiopathy, and pre-diagnosed dementia as covariates in our multiple regression analysis (Supplementary Table 2). This confirmed delirium’s significant association with elevated NFL levels, independent of comorbidities.

In contrast, a significantly more frequent occurrence of non-severe PNP was found in the control cohort, which may be due to the process of patient recruiting. Data on healthy elderly individuals who received CSF analysis without any neurological indication were not available. We therefore tried to identify neurological indications for lumbar puncture that were part of a diagnosis for which we would not expect an increase in the studied biomarkers.

sTREM2 and NPTX2 are debated biomarkers in delirium and AD research. Our study found no link between CSF sTREM2 or NPTX2 levels and AD, contrasting with studies showing mixed results. For instance, a study using ELISA, similar to our study, found no CSF sTREM2 correlation with AD (58). In two independent cohorts, the authors reported mean sTREM2 concentrations of 4,800 pg/mL and 3,800 pg/mL, respectively, in AD patients and 4,400 pg/mL and 3,200 pg/mL, respectively, in controls (58). In our study, we reported mean concentrations of 3,102 pg/mL in AD patients and 3,134 pg/mL in non-AD controls. Conversely, a mass spectrometry study reported significantly elevated CSF sTREM2 levels in AD patients (19), with mean concentrations of 231.2 pg/mL for AD patients and 195.6 pg/mL for controls, highlighting the possible variations due to measurement methods. Future biomarker research should compare different measurement methods and evaluate their accuracy.

Overall, our findings add to the growing body of evidence that neurodegeneration is one of the key aspects of the pathophysiology of delirium. NFL and tTau emerged as the most promising biomarkers; however, this does not automatically lead to clinical usage, for which the identification of a biomarker that could reliably distinguish between delirium and dementia would be more relevant. It also remains to be clarified whether neurodegeneration is a trigger for delirium, a consequence of neuroinflammation commonly associated with both delirium and neurodegeneration or a combination of these factors. Additionally, SNAP-25 and NPTX2 did not show promise as biomarkers for synaptic dysfunction in delirium, while current hypotheses suggest synaptic dysfunction plays a key role in the development of delirium. Therefore, further investigation into other potential biomarkers in this area, especially in prospective-designed studies, is highly warranted.

## 5 Limitations

The primary limitations of the preliminary data presented in this article, stemming from its retrospective nature, include potential disparities in disease severity between groups and a small and uneven study population. Retrospective data collection also prevented us from performing follow-up measurements to investigate the course of the biomarkers after resolution of delirium and a potential correlation with the outcome of delirium, the latter of which would be of particular clinical interest. The diagnosis of delirium was performed by neurology specialists and was coded in the discharge letter. However, it was not defined in the file if or which protocol led to the diagnosis of delirium. We therefore critically reviewed the medical file and the daily visit entries to determine whether the diagnosis was conclusive and met ICD-10 criteria. While this limitation certainly limits replicability, one strength of the retrospective study lies in the use of real-world data, reducing artificial settings.

Additionally, while comparing comorbidities across the three cohorts, we observed significant differences regarding systemic infections and neurological comorbidities. A minor limitation might also be the non-significant differences in age (Table 1). Based on the results of a study that found no association between NFL and the severity of pneumonia (49), it appears that the mere presence of infections did not skew the NFL levels in our delirium cohort. Our analysis also demonstrates that the associations between NFL and delirium remain robust after correcting for significant differences in cortical atrophy, cerebral microangiopathy, pre-diagnosed dementia, and non-significant age differences (Supplementary Table 2).

BMI was only assessed in 5 out of 16 patients. Serum NFL has been shown to be negatively correlated with BMI (59), which we could not correct.

To overcome the limitations inherent in retrospective data collection and to reduce the potential impact of confounding comorbidities, conducting a validation study with a prospective, case-matched design is essential.

## Data availability statement

The datasets presented in this article are not readily available because for data protection reasons, the pseudonymized primary data cannot be made publicly accessible in accordance with German data protection regulations. Requests to access the datasets should be directed to JP, johannes.piel@uksh.de.

## Ethics statement

The studies involving humans were approved by the Ethics Committee of Kiel University, Kiel, Germany. The studies were conducted in accordance with the local legislation and institutional requirements. Written informed consent for participation in this study was provided by the participants or their legal guardians/next of kin.

## Author contributions

JP: Conceptualization, Data curation, Formal analysis, Funding acquisition, Investigation, Methodology, Project administration, Supervision, Validation, Visualization, Writing – original draft, Writing – review & editing. LB: Data curation, Formal analysis, Investigation, Methodology, Visualization, Writing – original draft, Writing – review & editing. FL: Conceptualization, Data curation, Formal analysis, Investigation, Methodology, Resources, Supervision, Writing – original draft, Writing – review & editing. K-PW: Formal analysis, Investigation, Methodology, Resources, Writing – original draft, Writing – review & editing. JD: Data curation, Formal analysis, Investigation, Project administration, Resources, Supervision, Validation, Visualization, Writing – original draft, Writing – review & editing.
