# Supplementary material for: Serum NFL and tau, but not serum UCHL-1 and GFAP or CSF SNAP-25, NPTX2, or sTREM2, correlate with delirium in a 3-year retrospective analysis
Source: Front Neurol. 2024 Mar 19;15:1356575. doi: 10.3389/fneur.2024.1356575 (PMC10985356; doi:10.3389/fneur.2024.1356575)
Supplement: Supplementary file 1 [file Data_Sheet_1.docx]

Supplementary Material

Neurofilament light chain and Tau, but not UCHL-1, GFAP, SNAP-25, NPTX2 or sTREM2 correlate with delirium in a 3-year retrospective analysis

**Supplementary Table 1.** ICD codes for recruitment.

| Delirium [N = 306] | ICD E16.1, E51.2, F05.0, F05.1, F05.8, F05.9, G92, G93.4 and I67.4 |
| --- | --- |
| Controls [N = 491] | ICD F44.3, F44.4, F44.5, F44.6, F44.88, F45.0, F45.38, F45.40, F45.41, F45.8, F45.9, G43, G43.1, G43.2, G43.3, G44.0, G44.2, G44.4, G44.8, G91.20 and G93.2 |
| AD [N = 155] | ICD F00.0, F00.1, F00.2 and F00.9 |

**Supplementary Table 2.** Multiple regression analysis of serum NFL levels (dependant variable) with delirium, age, cortical atrophy, cerebral microangiopathy and pre-described dementia as independent variables. Serum NFL levels were transformed using the natural logarithm to achieve normal distribution of residuals. Regression coefficient β is reported in logarithmic units of serum NFL and with 95% confidence interval. R^2^ = 0.2645.

|  | β (95% CI) | p-value |
| --- | --- | --- |
| Delirium | 0.8087 (0.3792 to 1.238) | 0.0004* |
| Age | 0.0080 (- 0.0118 to 0.0278) | 0.4230 |
| Cortical atrophy | 0.4943 (0.1291 to 0.8595) | 0.0088* |
| Cerebral microangiopathy | - 0.2661 (- 0.6635 to 0.1313) | 0.1858 |
| Pre-described dementia | - 0.2334 (- 0.7329 to 0.2661) | 0.3542 |


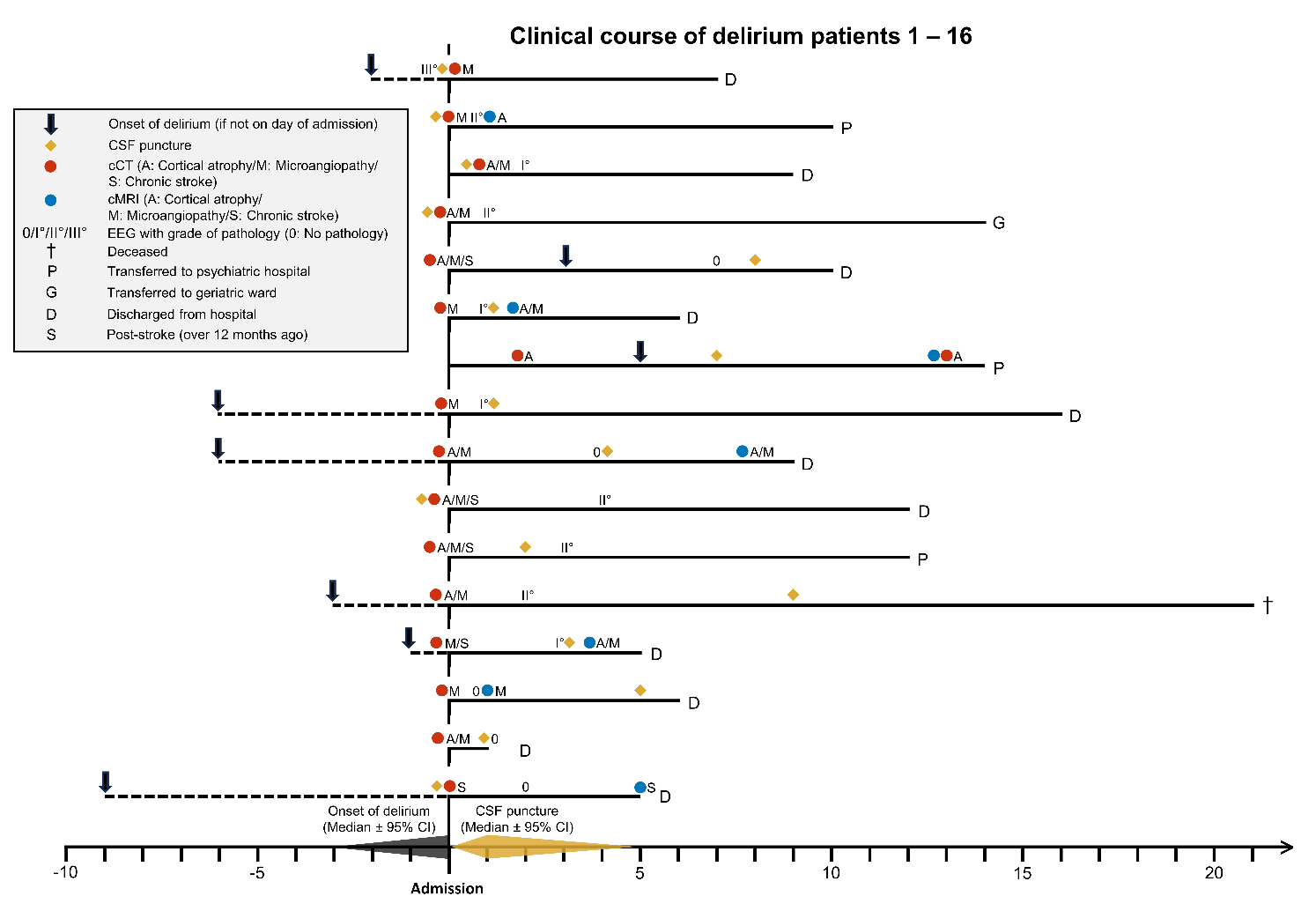


**Supplementary Figure 1.** Clinical course of delirium patients 1-16.


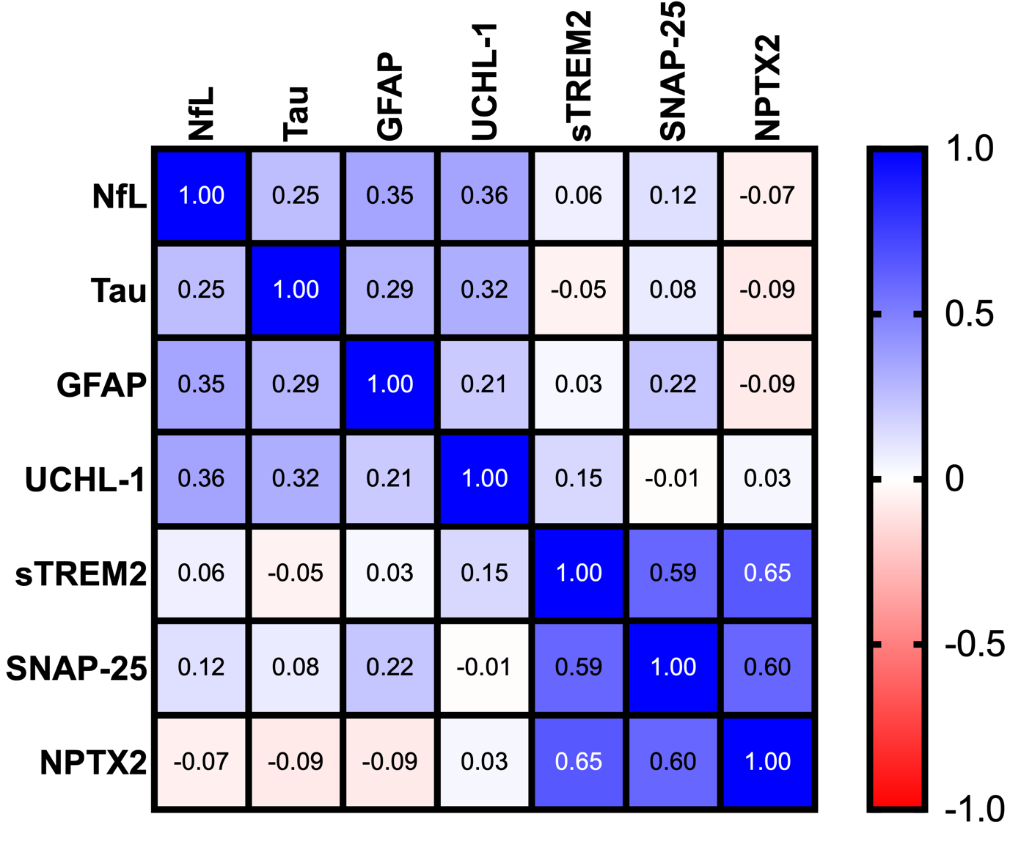


**Supplementary Figure 2.** Heatmap of Spearman’s ρ for correlations of serum (NFL, tau, GFAP, UCHL-1) and CSF (sTREM2, SNAP-25, NPTX2) biomarker levels in delirium, AD and controls.
